# Sexual orientation and all-cause mortality: A population-based prospective cohort study in southern Sweden

**DOI:** 10.1016/j.puhip.2020.100032

**Published:** 2020-07-12

**Authors:** Martin Lindström, Maria Rosvall

**Affiliations:** a Social Medicine and Health Policy, Department of Clinical Sciences, Malmö University Hospital, Lund University, S-205 02, Malmö, Sweden; b Department of Community Medicine and Public Health, Sahlgrenska Academy, Institute of Medicine, University of Gothenburg, Sweden

**Keywords:** Sexual orientation, LGBT, Bisexual, Homosexual, Mortality, Sweden

## Abstract

**Objectives:**

To investigate associations between sexual orientation and all-cause mortality.

**Study design:**

Prospective cohort study.

**Methods:**

The 2008 public health survey in Scania was conducted with a postal questionnaire later linked to 9.3-year prospective death register data, including 25,071 respondents, aged 18–80. Analyses were conducted with sex-stratified survival analyses.

**Results:**

In the models including age, birth country and socioeconomic status, bisexual men had a hazard rate ratio (HRR) 1.91 (1.10–3.30) compared to heterosexual men, and bisexual women had a HRR 3.18 (1.64–6.18). No significant differences were observed for homosexuals. Other women had a HRR 2.32 (1.47–3.67).

**Conclusions:**

Bisexuals men and women had higher mortality than heterosexuals.

## Highlights

• Studies on sexual identity and mortality are scarce and mostly from the USA.
• Most US studies concern homosexual men, far fewer minority women and bisexuals.
• Bisexual men and women had higher prospective mortality than heterosexuals.
• Mortality among homosexual men and women did not significantly differ from heterosexuals.
• Other women had significantly higher mortality than heterosexual women.

Sexual minorities, i.e. LGBT ​+ ​or non-heterosexuals, have often been shown to have higher mortality [1], morbidity and physical health [2] than heterosexuals.

Most studies investigating the association between sexual orientation and mortality have been conducted in the USA. In a US study, sexual minority 17-59-year old men and women were observed to have higher mortality [1] than heterosexuals. In another US study, homosexual women had higher suicide-mortality than heterosexual women, while suicide mortality for homosexual men did not significantly differ from heterosexual men. In the same study, all-cause mortality for homosexual men and women did not significantly differ from all-cause mortality for heterosexuals [3]. In a third US study, homosexual women had significantly higher rates of suicide and breast cancer mortality but not all-cause-mortality [4]. In most US studies, sexual minorities have mostly either been investigated as an aggregated group, or only homosexuals have been investigated as a separate group. Far fewer studies have been conducted regarding sexual minority LGBT ​+ ​people in Europe, and fewer studies have been conducted regarding bisexual men and women as well as other sexual minorities. Furthermore, more studies are still needed to establish whether increased levels of risk factors and behavioral differences compared to heterosexuals may also be reflected in higher mortality [1].

The comparatively small numbers of sexual minorities even in large population-based studies based on random samples of the population have resulted in the use of statistical imputation techniques to compensate for substantial numbers of internally missing. However, imputation may also incur statistical mistakes and, thus, mistaken conclusions [5]. The present large, population-based, prospective study investigates homosexual, bisexual and other men and women in Sweden, using mostly covariates from register data with no internally missing values.

The aim of this study was to investigate associations between sexual orientation and mortality using survival (Cox regression) analyses, adjusting for age, country of birth and socioeconomic status (SES), and stratifying for sex.

## 1 Study population

The public health survey in Scania 2008 is cross-sectional, and based on a stratified sample of the official register population aged 18–80 in Scania, located in the southernmost part of Sweden. A postal invitation letter including a questionnaire was sent, subsequently followed by three reminders to non-respondents. The possibility to answer the questionnaire online was also given. A sum of 28,198 responded, which yielded a 54.1% participation rate.

This study was approved by the Ethical Committee (*Etikprövningsnämnden*) in Lund (No. 2010/343).

## 2 Dependent variable

Mortality was followed prospectively from 27 August-14 November 2008 (registration date of individual answers) until 31 December 2017 (9.3 years later), or until death (1484 respondents of 25,071 in the present study). A total of 11,378 men and 13,693 women were included, excluding 3991 respondents with missing values on any of the variables (sexual orientation and socioeconomic status from the baseline postal questionnaire) in this study (another 136 respondents of the original 28,198 respondents were lost to follow-up). All analyses were restricted to these 25,071 respondents. The Swedish ten-digit person number system makes it possible to connect baseline data from the 2008 survey with the Swedish national cause of death register (the Swedish National Board on Health and Welfare) by a third party (private company). The person numbers were deleted before delivery.

## 3 Independent variables

*Sexual orientation* was assessed with the item “Do you regard yourself today as heterosexual, bisexual, homosexual (as phrased in the questionnaire), or other?“.

All analyses were stratified by *sex*.

*Age* was included as a continuous variable.

*Country of birth* was defined as born in Sweden and born in any other country.

*Socioeconomic status (SES)* included non-manual employees in higher, medium and lower positions, skilled and unskilled manual workers, and self-employed/farmers. The categories outside the workforce included unemployed, students, early retired (before 65), on long-term sick leave, pensioners aged 65-, and unclassified.

Prevalence (%) of all variables were calculated stratified by sex (not in tables). Hazard rate ratios (HRR:s) with 95% confidence intervals (95% CI:s) of mortality according to variable at baseline were estimated in bivariate survival (Cox-regression) models adjusted for age (not in tables). Multiple survival (Cox-regression) models were applied to calculate associations between sexual orientation and mortality adjusted for age (model 1), age and country of birth (model 2), and age, country of birth and SES (model 3), comparing the sexual minorities bisexuals, homosexuals and other with heterosexuals (Table 1). In order to conduct calculations, the SPSS software version 25.0 was used.

**Table 1:** Age-adjusted and multiple adjusted hazard rate ratios (HRR, 95% CI) of all-cause mortality according to sexual orientation. The public health survey in Scania 2012. N ​= ​25,071 (11,378 men and 13,693 women).

Sexual orientation	Men		
	HRR (95% CI)^a^	HRR (95% CI)^b^	HRR (95% CI)^c^
Heterosexual	1.00	1.00	1.00
Bisexual	1.91 (1.10–3.31)	1.91 (1.11–3.31)	1.91 (1.10–3.30)
Homosexual	0.88 (0.40–1.97)	0.88 (0.40–1.97)	0.85 (0.38–1.90)
Other	2.20 (1.44–3.36)	1.28 (0.84–1.96)	1.28 (0.84–1.95)

**Sexual orientation**	**Women**		
	**HRR (95% CI)**^a^	**HRR (95% CI)**^b^	**HRR (95% CI)**^c^
Heterosexual	1.00	1.00	1.00
Bisexual	3.58 (1.85–6.92)	3.43 (1.77–6.65)	3.18 (1.64–6.18)
Homosexual	2.17 (0.90–5.24)	2.13 (0.88–5.13)	2.15 (0.89–5.19)
Other	2.39 (1.51–3.78)	2.37 (1.50–3.74)	2.32 (1.47–3.67)

a Adjusted for age.

b Adjusted for age and country of birth.

c Adjusted for age, country of birth and socioeconomic status (SES).

Among men included in the study, 96.2% were heterosexuals, 1.4% bisexuals, 1.0% homosexuals and 1.4% other. Among women, 96.5% reported heterosexual orientation, 2.0% bisexual, 0.7% homosexual and 0.9% other orientation. The distributions according to age, country of birth and SES have been reported previously from this public health survey (not shown in table) [6].

Age-adjusted HRRs for those born outside Sweden compared to those born in Sweden were 0.99 (0.80–1.24) and 1.25 (0.98–1.58) for men and women, respectively. Among men, age-adjusted HRRs were significantly higher for lower level non-manual employees, skilled and unskilled manual workers, the unemployed, students and respondents on long-term sick-leave compared to non-manual employees in higher positions. Among women significantly higher HRRs were only observed for the unemployed and old-age pensioners compared to non-manual employees in higher positions (not shown in table).

Bisexual men had consistently significantly higher HRRs than heterosexual men throughout the analyses, and the HRR was 1.91 (1.10–3.30) in the final model. Bisexual women also had significantly higher HRRs than heterosexual women, and the HRR in the final model was 3.18 (1.64–6.18). In contrast, the HRRs for homosexual men and women did not significantly differ from the heterosexual reference group. Homosexual men had a HRR 0.85 (0.38–1.90) in the final model, and homosexual women 2.15 (0.89–5.19). Men in the other sexual orientation group had HRRs that were consistently not statistically significant in models 1–3. In the final model, the HRR for men with other orientation was 1.28 (0.84–1.95). Women in the other group had statistically significant HRRs throughout the analyses, with HRR 2.32 (1.47–3.67) in the final model.

## 4 Discussion

Bisexual men and women had significantly higher HRRs than heterosexual men and women, respectively. In contrast, no statistically significant differences in HRRs were observed for homosexual men and women. Women but not men with other sexual orientation also had a significantly higher HRR than heterosexual women.

The higher mortality for bisexual men and women, but not for homosexual men and women, compared to heterosexual men and women, may be interpreted in terms of social conditions and legal and social development. Registered partnership was granted to same-sex couples in Sweden in 1995, and marriage in 2009. In fact, a previous study from Sweden based on register data from the 1996–2009 period has shown that same-sex couples, particularly men, had higher suicide rates than different-sex couples, i.e. during the period just before marriage was granted to same-sex couples [7]. The results of the present study may be viewed in light of the fact that the bisexual group is probably currently more vulnerable due to less social change in recent decades, as well as less change in attitudes towards their orientation [8]. One conclusion is that the results of this study may not be generalized directly to other countries. They should instead be related to the juridical and socio-cultural situation in the specific country studied. Other countries with legal and social change which differ from Sweden may e.g. have higher mortality for both bisexuals and homosexuals.

The study is population-based, large and has a prospective cohort design. The participation rate is similar to other studies in Sweden and other western countries. The representativeness of sociodemographic variables were satisfactory in the baseline survey with a consequently limited risk of selection bias [9]. This study is a population-based study with high statistical power and comparatively low proportion of internally missing, which makes it possible to avoid imputation. Mortality levels and patterns are in accordance with the general population in Sweden in the 2010–2012 regarding age and sex (not published). Specific diagnoses are difficult to analyze separately because of the low number of participants with sexual orientation other than heterosexual. Further studies are needed, and this data are planned to be extended prospectively. The age, sex and country of birth variables and the mortality data stem directly from the national population register and the national cause of death register, and have very high validity. The sexual orientation item has also been shown to have high validity, and the prevalence of sexual orientation other than heterosexual is similar to other studies [10].

## Declaration of competing interest

The authors declare that they have no conflicts of interests.
